# AI in Echocardiography: State-of-the-art Automated Measurement Techniques and Clinical Applications

**DOI:** 10.31662/jmaj.2024-0180

**Published:** 2024-12-06

**Authors:** Yukina Hirata, Kenya Kusunose

**Affiliations:** 1 Ultrasound Examination Center, Tokushima University Hospital, Tokushima, Japan; 2 Department of Cardiovascular Medicine, Nephrology, and Neurology, Graduate School of Medicine, University of the Ryukyus, Okinawa, Japan

**Keywords:** echocardiography, artificial intelligence, deep learning, automated measurements

## Abstract

The artificial intelligence (AI) technology in automated measurements has seen remarkable advancements across various vendors, thereby offering new opportunities in echocardiography. Fully automated software particularly has the potential to elevate the analysis and the interpretation of medical images to a new level compared to previous algorithms. Tasks that traditionally required significant time, such as ventricular and atrial volume measurements and Doppler tracing, can now be performed swiftly through AI’s automated phase setting and waveform tracing capabilities.

The benefits of AI-driven systems include high-precision and reliable measurements, significant time savings, and enhanced workflow efficiency. By automating routine tasks, AI can reduce the burden on clinicians, allowing them to gather additional information, perform additional tests, and improve patient care. While many studies confirm the accuracy and the reproducibility of AI-driven techniques, it is crucial for clinicians to verify AI-generated measurements and ensure high-quality imaging and Doppler waveforms to fully take advantage of the benefits from these technologies. This review discusses the current state of AI-driven automated measurements in echocardiography, their impact on clinical practice, and the strategies required for the effective integration of AI into clinical workflows.

## Introduction

Echocardiography plays a central role in the diagnosis and management of cardiovascular diseases, providing essential insights into cardiac morphology, motion, and function. The demand for echocardiographic examinations has significantly increased because of its expanding use in preoperative evaluations, heart failure management, and early detection of cardiomyopathies. This growing demand is coupled with the integration of advanced techniques, such as strain analysis and three-dimensional echocardiography, which not only offer detailed insights into the myocardial function and structure, but also add to the complexity and time required for examinations. According to the American Society of Echocardiography and the European Association of Cardiovascular Imaging guidelines, the examination time for comprehensive echocardiograms has increased by approximately 20% over the past decade ^(1)^. This increase is attributed to the need for more detailed evaluations, resulting in examination times that can exceed 30 min and place a significant burden on sonographers and clinicians, especially when dealing with high patient volumes. Despite these technological advancements, echocardiographic assessments remain heavily dependent on operator expertise, leading to variability in measurements and potential diagnostic errors ^(2)^. High daily workloads further exacerbate these challenges as clinicians strive to maintain accuracy within limited timeframes.

Thus, the growing need for efficient and accurate assessments has brought to the forefront the idea of using artificial intelligence (AI) as an aid in cardiac ultrasound imaging ^(3), (4), (5), (6), (7), (8), (9), (10), (11), (12), (13)^. By automating tasks traditionally performed by humans, AI can alleviate the burden on clinicians, thereby allowing them to use the saved time to gather additional information, respond flexibly to additional tests, and ultimately improve patient care ^(10)^. While numerous studies support the accuracy and the reproducibility of AI-driven techniques, clinicians must validate AI-generated measurements and ensure high-quality imaging and Doppler waveforms to maximize the benefits of these technologies. This review explores the current state of AI-driven automated measurements in echocardiography, their impact on clinical practice, and the strategies required for the effective integration of AI into clinical workflows.

## Automated Echo Interpretation

**Figure 1.**
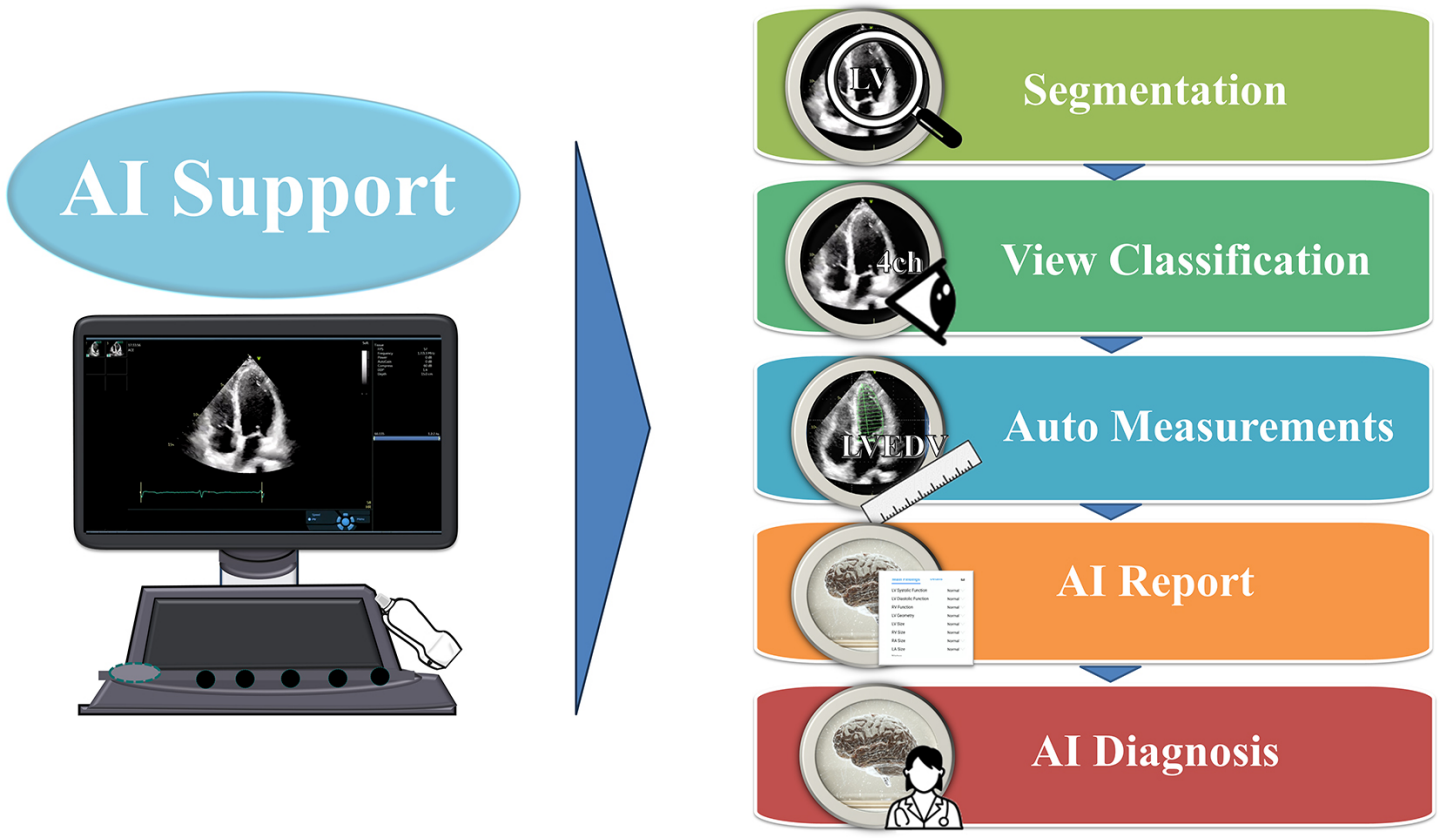
Illustration of the AI support in echocardiography workflows View Classification―AI identifies and labels the different views of the echocardiographic images. View Classification―AI classifies the images into specific types of echocardiographic views, such as apical 4-chamber (4ch). Auto Measurements―AI performs automatic measurements of cardiac structures, such as left ventricular end-diastolic volume (LVEDV). Echo AI Report―AI generates comprehensive echocardiographic reports based on automated measurements and analyses. AI Diagnosis―AI assists in diagnosing cardiac conditions by analyzing the echocardiographic data.

As illustrated in Figure 1, the AI support in echocardiography has evolved to include automated segmentation, view classification, auto measurements, and AI-generated reports and diagnoses ^(14), (15)^. Several promising studies using deep learning (DL) approaches have been published to classify standard echo views (e.g., apical and parasternal views), heart structure segmentation (e.g., ventricle, atrium, septum, myocardium, and pericardium), and cardiac disease prediction (e.g., heart failure, hypertrophic cardiomyopathy, cardiac amyloidosis, and pulmonary hypertension) in recent years ^(5), (8), (16), (17), (18)^. Additionally, several companies, such as TOMTEC Imaging Systems GmbH (Munich, Germany) and Ultromics (Oxford, United Kingdom), have already obtained a premarket Food and Drug Administration clearance for auto ejection fraction (EF) and echo strain packages using AI. Furthermore, US2.ai, Singapore provides not only automatic measurements, but also generates reports by determining normal or abnormal findings based on the latest guideline criteria. These fully automated analysis systems exemplify the potential of AI in echocardiography, demonstrating their ability to provide high-precision and reliable measurements, significantly reduce the examination time, and enhance the workflow efficiency. The list of companies and their provided AI tools is shown in Table 1.

**Table 1.** List of Commercial Software Packages Providing Automated Measurements or Diagnosis.

Company	Software package	AI-empowered tools
Siemens Medical Solutions Inc., USA	syngo Auto Left Heart, Acuson S2000 US System	Auto EF, Auto LV and LA volumes, Auto Strain for manually selected views
GE Healthcare, Inc., USA	Ultra Edition Package, Vivid Ultrasound Systems	Auto EF, Auto LV and LA volumes, Auto Strain for manually selected views
TOMTEC Imaging Systems GmbH, Germany	Tomtec-Arena/Tomtec-Zero	Auto EF, Auto LV and LA volumes, Auto Strain for manually selected views
Ultromics Ltd., United Kingdom	Echo Go/Echo Go Pro	Auto EF, Auto LV and LA volumes, Auto Strain, Auto identification of CHD (fully automated)
Dia Imaging Analysis Ltd., Israel	DiaCardio’s LVivoEF Software/LVivo Seamless	Auto EF and Auto Standard Echo View Identification (fully automated)
Caption Health, Inc., USA	The Caption Guidance Software	AI tool for assisting to capture images of a patient’s heart
Butterfly Network, USA	Butterfly Garden	Auto EF, Auto Standard Echo View Identification, etc.
US2.ai, Singapore	US2.ai	Auto Standard Echo and Strain (fully automated)
		Report generation based on guideline criteria

## Precision and Reliability in Echocardiographic Measurements

Accurate echocardiographic assessments are crucial for clinical decision-making, yet traditional manual methods often result in a variability caused by differences in operator experience and measurement techniques. For example, left ventricular ejection fraction (LVEF) assessments are subjective and influenced by observer experience, consequently leading to inconsistencies across institutions with readers of varying experience levels ^(19), (20)^. Interventions, such as teaching programs, have shown some success in reducing this variability ^(21), (22), (23)^, particularly for less experienced readers ^(24)^. However, variability can occur not only between different cardiologists, but also within the same observer’s repeated readings.

AI systems offer a solution by automatically tracing the endocardial borders to calculate the ejection fraction with minimal user intervention, ensuring reproducibility and reliability across different operators and institutions ^(8), (12), (25), (26)^. A recent study by Olaisen et al. ^(27)^ validated the accuracy of AI-driven measurements of the LV volume and LVEF in real-time and large databases, demonstrating a superior test-retest variability in inter-observer scenarios and non-inferior variability in intra-observer scenarios. It showed excellent feasibility in large internal and external databases, showing a good agreement with reference measurements, particularly within the LV EF domain of 45%-60%. Another study by Dadon, Z. et al. ^(28)^ showed that an AI-based tool on a handheld ultrasound device (HUD) operated by medical students for LVEF assessment had a high correlation with a cardiologist visual assessment. The AI-based LVEF measurement of the students’ HUD-acquired clips reached an agreement that was significantly higher than the student visual evaluation and almost as good as that of the cardiologists. The accuracy of Doppler indices was well documented in various studies. When compared with an expert echocardiographic reader, the automated measurements showed an excellent correlation (correlation coefficients for all measurements > 0.9) ^(10), (12), (29)^. Additionally, a study by Tromp et al. ^(30)^ validated a deep learning-based automated workflow against a large dataset, depicting that the AI system performed on par with expert cardiologists, offering precise measurements with reduced variability. Moreover, Mor-Avi, V. et al. ^(31)^ also reported that the DL-assisted approach significantly reduced the inter-reader variability in the measurement of the echocardiographic parameters of left-heart size and function, which were previously subjected to a considerable variability with conventional manual measurements. However, while the DL algorithm provides accurate automated measurements in most cases, they emphasized the necessity of the manual corrections by expert readers to optimize the diagnostic accuracy for all patients, highlighting the essential role of quality control by skilled readers.

## Precision and Reliability in Strain Imaging

Strain imaging, particularly two-dimensional (2D) speckle-tracking echocardiography, is another sophisticated technique used to assess myocardial deformation. This modality provides detailed insights into the myocardial function, detecting subtle changes that may not be apparent with conventional echocardiography. AI algorithms enhance the precision and the reliability of strain measurements by automatically identifying and tracking the myocardial speckles throughout the cardiac cycle ^(29), (32)^. A study conducted by Salte et al. ^(33)^ demonstrated that AI could significantly reduce inter- and intra-observer variabilities, enhancing the measurement consistency across different operators. The application of DL models, such as U-net, for the automatic segmentation of the LV resulted in high accuracy and reproducibility of strain measurements. These AI-driven models effectively standardized measurements, reducing potential human error and improving the overall diagnostic accuracy. AI mitigates inter-observer bias and inter-vendor variability, which are the significant challenges in manual strain measurements. For example, the EchoGo software from Ultromics has been validated to eliminate variability and user bias, ensuring consistent and accurate strain measurements, regardless of the operator’s experience level. This standardization is crucial for reliable patient monitoring over time and across different clinical settings.

**Figure 2.**
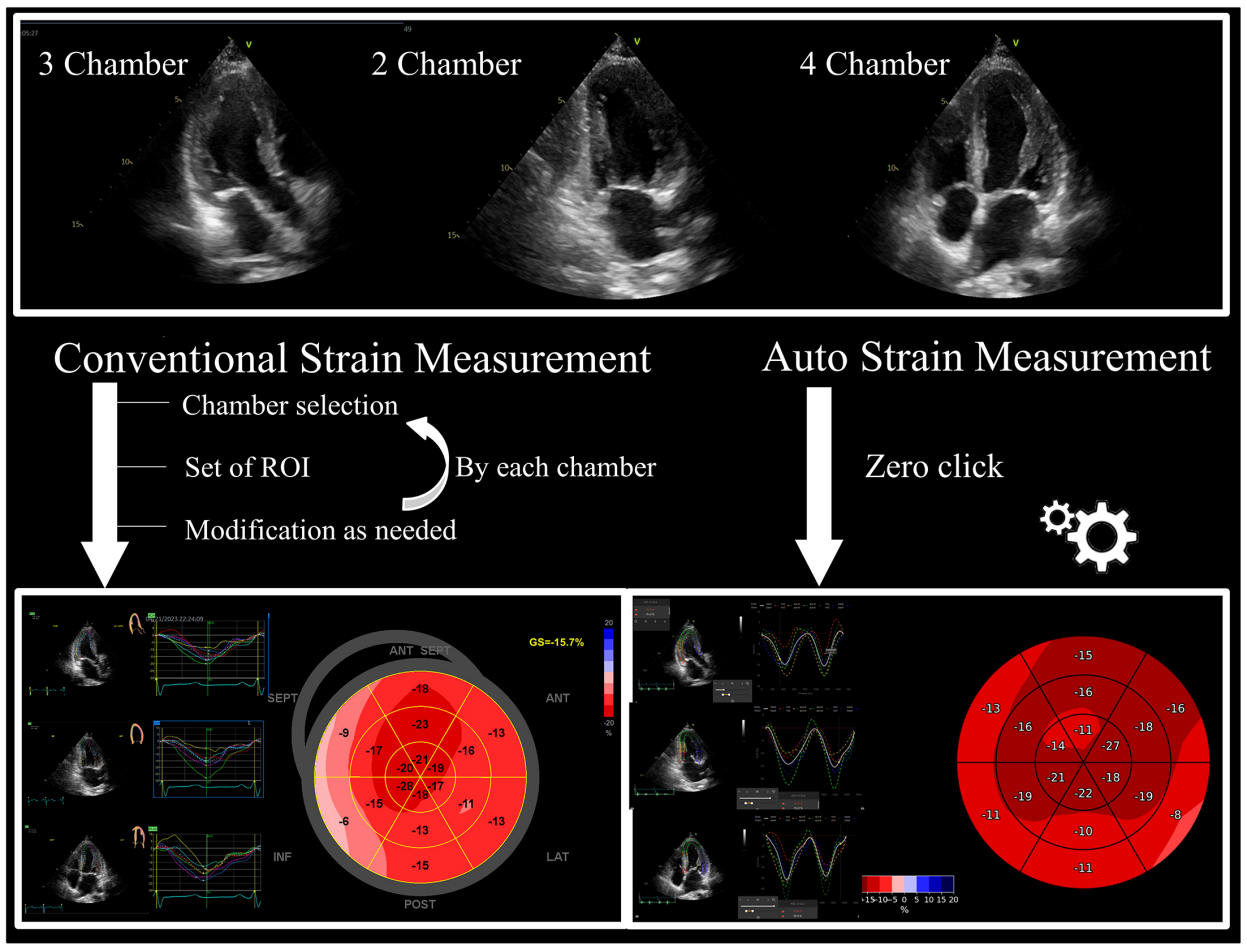
Comparison between conventional and automated strain measurements in echocardiography Conventional measurement involves manual steps like chamber selection and ROI modification (left). Automated measurement (right) requires no manual intervention and provides instant results. ROI: region of interest.

Our institution conducted a study involving 150 patients with varying EF values to further validate the accuracy and the efficiency of AI in strain measurements. Echocardiographic assessments were performed using various ultrasound machines. Strain measurements were compared between US2.ai, a fully automated DICOM reading software, and human experts using vendor-independent analysis software (EchoPAC ver.204). Figure 2 illustrates how an AI-driven strain measurement requires no manual clicks, unlike conventional methods that involve selecting chambers, displaying the ROI, and modifying the ROI. This efficiency is attributed to AI’s ability to automate the entire measurement process, from image selection to analysis, without requiring manual intervention ^(5), (8), (16), (34), (35)^. The DL-based workflow for the LV strain consists of two modules: the conventional 2D echo module and the strain module. The 2D module uses convolutional neural network (CNN)-based classifiers to categorize echocardiographic DICOM files into 2D videos, which are then classified into A4C, A2C, or A3C views, automatically excluding low-quality images. High-quality clips are analyzed with a CNN model to trace the LV endocardial border for each frame, identifying cardiac cycles from end-diastole to end-systole and calculating linear measurements and chamber volume ^(12)^. The strain module measures the circumferential lengths of the traced endocardium for each frame, creating drift-corrected strain curves based on the identified cardiac cycle. The final global longitudinal strain (GLS) value is the average of all cycles on the highest-quality video selected by the algorithm, ensuring the most accurate measurements. This system does not average all available videos, but selects that with the highest confidence to maximize accuracy. This entire process allows for automatic strain measurements without manual clicks or adjustments. Our study found a significant correlation between human and AI measurements across various echocardiographic parameters. For GLS, the AI measurements showed a strong positive correlation with the human expert measurements. The Bland-Altman plot demonstrated a good agreement. For the LA strain, the AI measurements also correlated well with the human measurements, although AI tended to slightly underestimate the LA strain compared to human experts.

## Time Efficiency

Table 2 shows the time efficiency of the automated echocardiographic methods. The AI technology in automated measurements has seen remarkable advancements across various vendors. Tasks that traditionally required significant time, such as ventricular and atrial volume measurements and Doppler waveform tracing, can now be performed swiftly through AI’s automated phase setting and waveform tracing capabilities ^(36)^. In the strain analysis, AI facilitates the immediate recognition of necessary cross-sections and initiates tracing without a manual input, significantly reducing the workload on clinicians ^(33), (34)^. Shiokawa’s study highlighted that using the Philips EPIQ CVx system, AI-enabled measurements allowed beginners to achieve the same efficiency as experienced echocardiographers ^(37)^. The study involved a range of operators with different experience levels and found that AI-driven measurements not only enhanced the time efficiency, but also significantly reduced the variability between users. The ability of AI to provide consistent and reliable results across different skill levels underscores its potential to streamline echocardiographic assessments and improve workflow. Olaisen et al. ^(27)^ reported that an AI-assisted examination significantly reduced the total acquisition and processing time by 77% (from 7′30″ to 1′54″) without compromising accuracy. Results on the automatic measurements of the LV volumes and EF by AI further support these findings.

**Table 2.** Time Efficiency of Automated vs. Manual Echocardiographic Methods.

Authors	Year	Target	Measurement	Vendor	Manual/automated measurement time	Time saved (%)	Notes
Knackstedt et al.^(16)^	2015	255 patients	EF and LS	TomTec	Manual: Not specified	-	The fully automated system provided rapid and reproducible EF and LS measurements with 0% variability in automated measurements. Good agreement with manual methods was observed.
					Automated: 8 ± 1 s		
Lang et al.^(10)^	2021	200 subjects	16 parameters LVDd, LVDs, IVS, LVPW, LVOTd, LVOT-VTI, LVEDV (A2C, A4C), LVESV (A2C, A4C), LAV (A2C, A4C), E, A, e’ (sep, lat),	CNN model	Average	41%	Reduced the variability of most parameters to below 10%.
					11′33″/6′48″		
Mor-Avi et al.^(31)^	2023	12 subjects by ten experts	20 parameters LVDd, LVDs IVS, LVPW, LVOTd, LVOT-VTI, LVEDV (A2C, A4C), LVESV (A2C, A4C), EF, LAV (A2C, A4C), E, A, e’ (sep, lat)	Novel AI software developed collaboratively by TOMTEC	Average	43%	DL algorithm showed good agreement with reference technique. Manual revisions improved accuracy slightly. Significant reduction in inter-reader variability.
					12′00″/6′49″		
Olaisen et al.^(27)^	2024	50 consecutive patients	LVEDV, LVESV, and EF	Novel AI software (real-time application)	Median	77%	Test-retest reproducibility was superior in inter-observer scenarios and non-inferior in intra-observer scenarios. AI measurements showed good agreement with reference measurements in both real-time and large research databases.
					7′30″/1′54″		
Hirata et al.^(38)^	2024	23 consecutive patients with varying image quality and conditions by expert	30 parameters LVDd, LVDs IVS, LVPW, LVEDV (A2C, A4C), LVESV (A2C, A4C), LAV (A2C, A4C), EF, SV, LVOTd, E, A, DT, e’ (sep, lat), a’ (sep, lat), s’(sep, lat), TRV, TAPSE, TAM, LVOT VTI, LVOT peakV, RVOT peakV, AoVmax	US2.ai software	Average	51%	Significant time reduction observed, especially with a good image quality. Manual adjustments required for poor image quality.
					5′25″/2′39″		
Shiokawa et al.^(37)^	2024	30 consecutive patients	LVDd, LVDs IVS, LVPW, E, A, DT, e’ (sep, lat), a’ (lat), LVOT VTI, LVOT peakV	Philips Healthcare	Average	27.6%	AI significantly reduced measurement time for experts and beginners, less so for intermediates.
					1′22″/0′59″		

Furthermore, our study using US2.ai software found significant time savings in echocardiographic examinations ^(38)^. In a prospective, single-center pilot study involving 23 consecutive patients with complex heart disease, arrhythmias, or poor image quality, the manual measurements took an average of 5′25″, while the automated measurements reduced this time to only 2′39″, presenting a reduction of approximately 50%. Additionally, the use of this AI-driven software reduced the average overall examination time to 9′33″. This efficiency was largely caused by AI’s ability to automatically perform measurements during the exam, eliminating the need for sonographers to manually operate the panel as they proceed. By allowing clinicians to focus solely on acquiring the necessary images, the examination process becomes more efficient, significantly shortening the overall time required for each patient. The incorporation of AI into routine clinical practice not only speeds up the process, but also reduces the workload on sonographers, thereby significantly benefiting both clinicians and patients.

## AI for Diagnosis of Cardiovascular Diseases

Integrating AI into echocardiographic practices has significantly advanced diagnostic capabilities, particularly through the development of generative AI that can create comprehensive echocardiographic reports based on numerical data and established clinical guidelines. By referencing the guidelines from authoritative bodies, such as the ASE/European Association of Cardiovascular Imaging (EACVI), AI can draft reports that include diagnostic conclusions and recommended actions ^(39), (40), (41), (42)^. The use of US2.ai software has shown significant potential in improving diagnostic workflows. Our study demonstrated that US2.ai could substantially reduce report times while maintaining a high diagnostic accuracy ^(38)^. This software processes echocardiographic data in real time, automatically generating complete reports with minimal user intervention. By streamlining the report generation process, US2.ai enhances the efficiency of echocardiographic assessments, consequently allowing clinicians to deliver quicker and more reliable diagnoses. This advanced capability of AI to create accurate, guideline-based diagnostic reports not only enhances efficiency, but also ensures that patients receive timely and precise evaluations.

## AI’s Challenges in Echocardiographic Measurements

**Figure 3.**
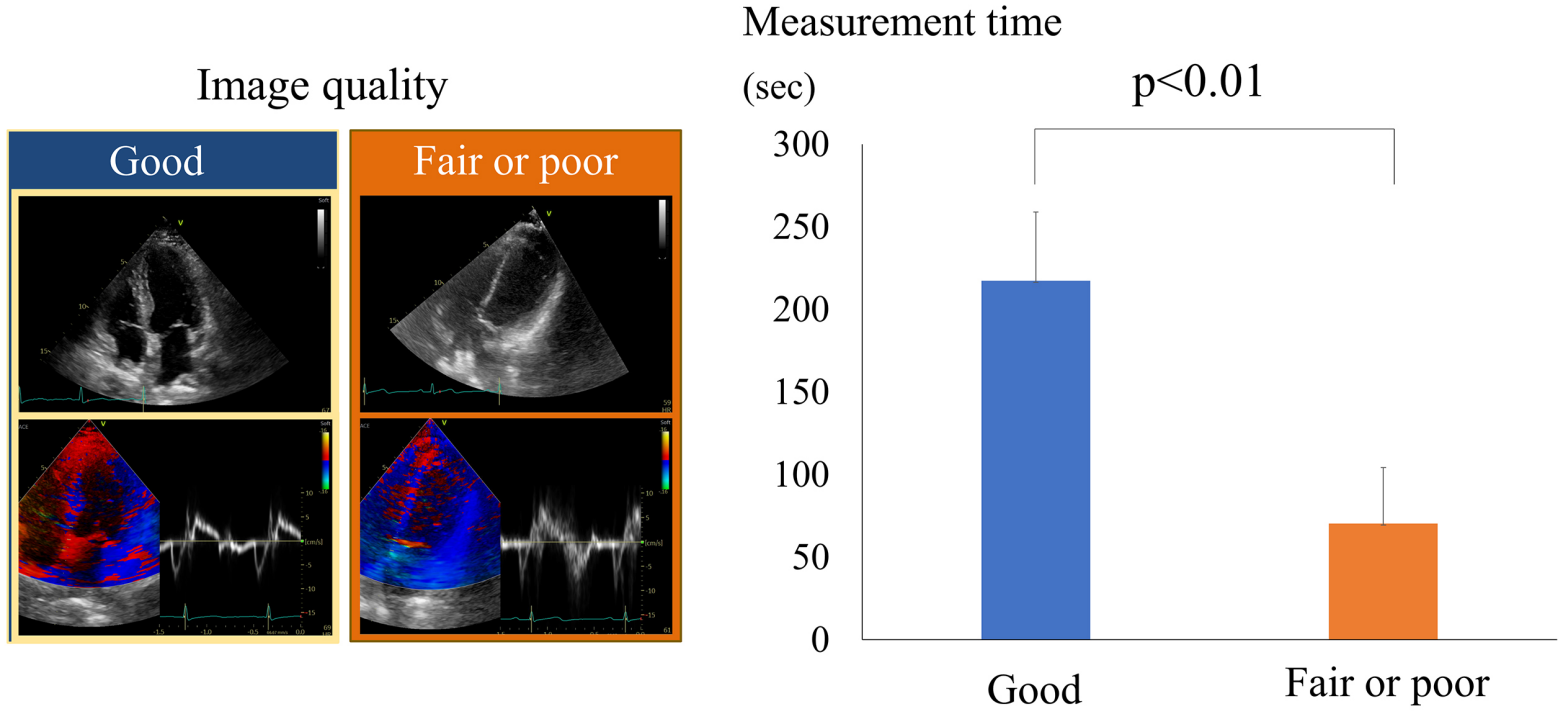
Impact of the image quality on the measurement time in echocardiography The left side shows examples of good-quality images compared to fair- or poor-quality images. The right-side graph demonstrates that fair- or poor-quality images require significantly more time for measurements (p < 0.01) compared to good-quality images, while reporting time remains consistent (ns).

While AI can significantly enhance echocardiographic workflows, challenges remain. One significant issue is the image quality. A poor image quality can lead to inaccurate results, even with AI. Figure 3 illustrates the impact that image quality has on the measurement time ^(38)^. Poor- or fair-quality images require more corrections, which significantly increases the measurement time compared to good-quality images. This is because AI measurements are often inaccurate with lower-quality images, necessitating more manual adjustments. Consequently, traditional manual measurements can sometimes be faster in these scenarios.

**Figure 4.**
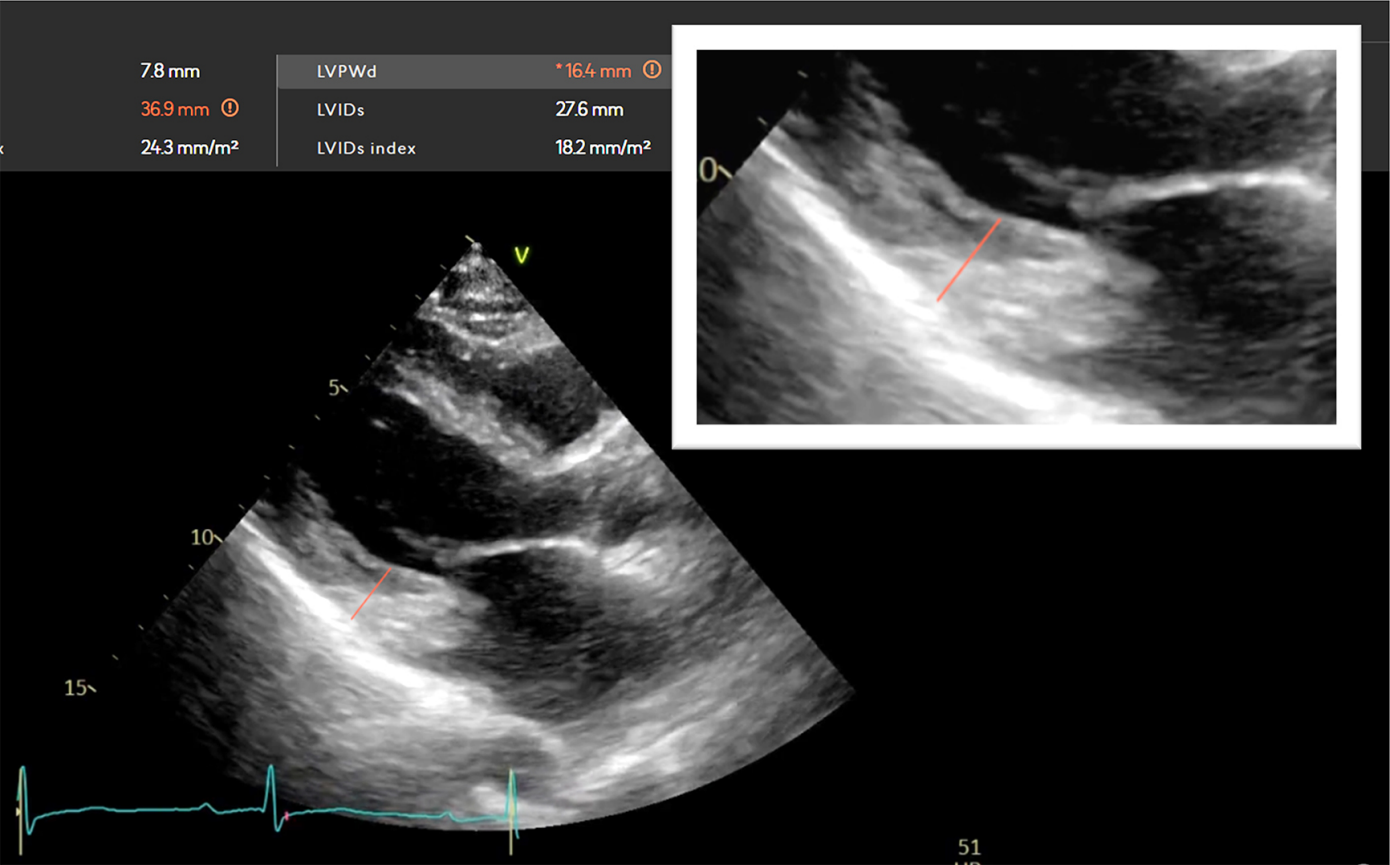
Erroneous automated measurement case of the posterior wall using AI Inclusion of chordae tendineae in the measurement results in a wall thickness overestimation (16.4 mm), potentially leading to a misdiagnosis of concentric hypertrophy. This case highlights the necessity for an accurate anatomical identification in AI-driven echocardiography.

Additionally, AI systems may struggle with distinguishing fine details, such as papillary muscles and chordae tendineae, which can affect the measurement accuracy. The measurements for the LV wall thickness (IVS and LVPW) were less consistent, highlighting the need for a careful assessment to avoid including irrelevant structures, such as the right ventricular zone or the subvalvular tissue of the tricuspid valve. Figure 4 demonstrates a measurement of the left ventricular posterior wall (LVPW) using AI, but this measurement includes the chordae tendineae, leading to a wall thickness overestimation. This overestimation can result in a misdiagnosis of concentric hypertrophy, highlighting a significant challenge in AI-driven echocardiography. Furthermore, no clear consensus exists on how to trace the endocardial border, and it may be difficult to differentiate between compact myocardium and trabeculations ^(40), (43)^. The initial version of the AI software was trained on the data from the open CAMUS data set, which included too much of the trabeculations and papillary muscles into the myocardium, leading to an LV volume underestimation ^(18)^. This highlights the importance of ensuring the representativeness of the training data and the challenges of the manual measurements of the LV volumes and EF. Additionally, MacKay, EJ et al. ^(44)^ highlighted that, in clinical environments, where consistently obtaining appropriate loop quality images is challenging (e.g., perioperative or emergency settings with point-of-care echocardiography performed by non-sonographers), AI reliability may be compromised. This underscores the importance of optimal image acquisition and the potential limitations of AI in less-controlled settings.

In conclusion, the accuracy of AI measurements heavily depends on the image quality and the correct anatomical identification. Therefore, operator expertise in obtaining optimal images and recognizing anatomical structures is crucial in ensuring reliable AI interpretations. This highlights the need for continuous training and supervision when integrating AI into clinical workflows to prevent diagnostic errors.

## Approaching Work Efficiency through AI Delegation

**Figure 5.**
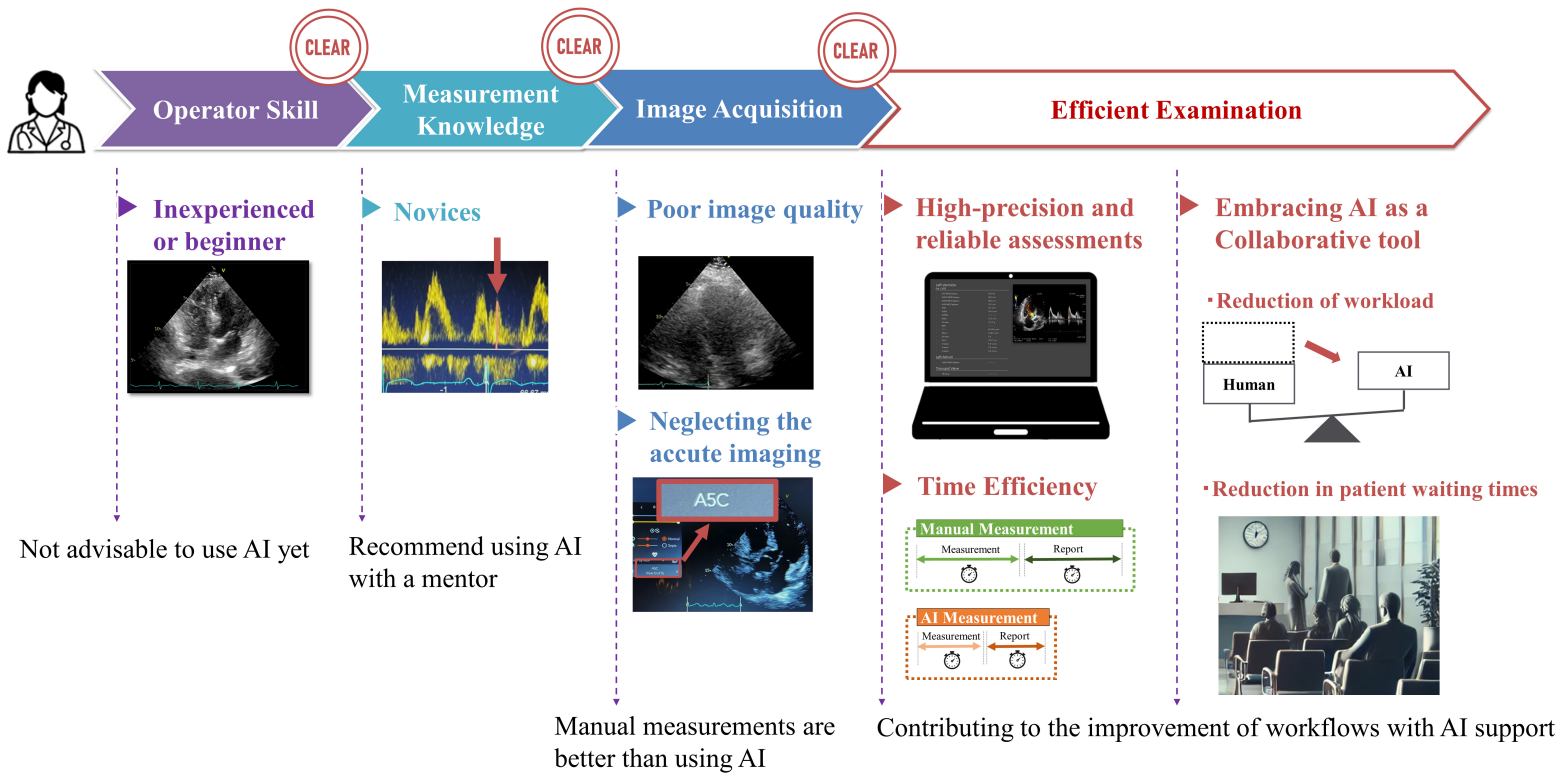
Strategies for the effective use of AI in echocardiography Inexperienced operators should not use AI alone, while novices are advised to use AI with a mentor. Manual measurements are better with a poor image quality or a neglected imaging. Once these challenges are addressed, high-precision and reliable assessments, improved time efficiency, and reduced workload and patient waiting times are achieved by integrating AI as a collaborative tool.

Integrating AI into echocardiographic workflows has shown a significant potential in enhancing the work efficiency. Figure 5 illustrates the effective strategies for leveraging AI to improve the workflow in echocardiography. It demonstrates the importance of operator skill and measurement knowledge in effectively using AI. Inexperienced operators might struggle with obtaining optimal cross-sectional images, which may lead to inaccurate measurements and potentially erroneous diagnoses when using AI-based tools. Therefore, it is crucial to ensure that operators are well-trained in image acquisition techniques. Correspondingly, inexperienced operators or beginners should avoid relying solely on AI because their lack of experience may lead to misinterpretations or overreliance on automated results. Instead, AI should be used with the guidance of a mentor to ensure the obtainment of accurate and reliable outcomes. For novices, AI can be a supportive tool when used alongside experienced supervision. The figure also highlights the impact of the image acquisition quality. A poor image quality can significantly hinder the effectiveness of AI-driven measurements, necessitating manual adjustments and potentially increasing the overall examination time. Therefore, it is essential to consistently strive for high-quality images to maximize the benefits of AI integration. AI’s effectiveness in the echocardiographic measurement heavily depends on the quality of the images captured, which, in turn, relies on the operator’s skill. High-quality image capture is essential for AI to perform accurate and reliable measurements.

Clearing these hurdles can significantly improve the workflow efficiency through AI usage. As discussed, AI can provide highly accurate and reproducible test results. It also allows for faster task completion. This leads to reduced workload for examiners and shorter waiting times for patients, making the entire examination process more efficient in several ways. By leveraging AI, echocardiographic workflows can achieve improved accuracy, efficiency, and overall quality of care.

## Conclusion

The integration of AI into echocardiography presents an opportunity of revolutionizing clinical workflows and patient care. The potential benefits of AI can be fully realized by addressing challenges related to operator skill and image quality and ensuring continuous training and education. AI has the capacity to deliver highly accurate and reproducible results, streamline tasks, and significantly reduce the workload and patient waiting times. However, the importance of human oversight remains paramount in validating AI-driven findings and ensuring the highest standards of care. As the AI technology continues to advance, clinicians must embrace these innovations and integrate them thoughtfully into practice, ultimately enhancing the efficiency and the effectiveness of echocardiographic assessments and patient outcomes.

## Article Information

### Conflicts of Interest

None

### Sources of Funding

This work was partially supported by JSPS Kakenhi grant numbers 21K12706 to Y. Hirata and 23K07509 to K. Kusunose and a grant from the Japan Agency for Medical Research and Development (AMED, JP22uk1024007) to K. Kusunose.

### Approval by Institutional Review Board (IRB)

None.
